# Arginine Supplementation Promotes Extracellular Matrix and Metabolic Changes in Keratoconus

**DOI:** 10.3390/cells10082076

**Published:** 2021-08-13

**Authors:** Tina B. McKay, Shrestha Priyadarsini, Tyler Rowsey, Dimitrios Karamichos

**Affiliations:** 1 Department of Cell Biology, University of Oklahoma Health Sciences Center, Oklahoma City, OK 73104, USA; TMCKAY@MGH.HARVARD.EDU; 2 Dean McGee Eye Institute, Oklahoma City, OK 73104, USA; shrestha.priyadarsini@gmail.com (S.P.); tylergrowsey@gmail.com (T.R.); 3 North Texas Eye Research Institute, University of North Texas Health Science Center, Fort Worth, TX 76107, USA; 4 Department of Pharmaceutical Sciences, University of North Texas Health Science Center, Fort Worth, TX 76107, USA; 5 Department of Pharmacology and Neuroscience, University of North Texas Health Science Center, Fort Worth, TX 76107, USA

**Keywords:** arginine, collagen, cornea, metabolomics, extracellular matrix, keratoconus, hydroxyproline, tissue-engineered cornea

## Abstract

Keratoconus (KC) is a common corneal ectatic disease that affects 1:500–1:2000 people worldwide and is associated with a progressive thinning of the corneal stroma that may lead to severe astigmatism and visual deficits. Riboflavin-mediated collagen crosslinking currently remains the only approved treatment to halt progressive corneal thinning associated with KC by improving the biomechanical properties of the stroma. Treatments designed to increase collagen deposition by resident corneal stromal keratocytes remain elusive. In this study, we evaluated the effects of arginine supplementation on steady-state levels of arginine and arginine-related metabolites (e.g., ornithine, proline, hydroxyproline, spermidine, and putrescine) and collagen protein expression by primary human corneal fibroblasts isolated from KC and non-KC (healthy) corneas and cultured in an established 3D in vitro model. We identified lower cytoplasmic arginine and spermidine levels in KC-derived constructs compared to healthy controls, which corresponded with overall higher gene expression of arginase. Arginine supplementation led to a robust increase in cytoplasmic arginine, ornithine, and spermidine levels in controls only and a significant increase in collagen type I secretion in KC-derived constructs. Further studies evaluating safety and efficacy of arginine supplementation are required to elucidate the potential therapeutic applications of modulating collagen deposition in the context of KC.

## 1. Introduction

Arginine is a conditional essential amino acid important in protein synthesis and signal transduction in mammalian cells [1]. Like many amino acids, arginine can be absorbed from the diet or biosynthesized from glutamate depending on the nutritional status of the organism [2,3]. Metabolism of arginine occurs primarily through the urea cycle, which involves conversion of arginine to ornithine, citrulline, and ultimately urea. Arginine plays a fundamental role in the production of many other metabolites, including the polyamines—spermidine and putrescine—that function in regulating cell proliferation and DNA replication [4]. Arginine is an important metabolite that can also serve as a precursor to proline and hydroxyproline, which are both highly present in collagen monomers. Collagen structure consists of repeating motifs of X-Y-glycine, with X usually proline and Y as hydroxyproline [5]. Arginine is also a precursor to creatine which is converted to phosphocreatine as an energy storage molecule primarily in skeletal muscle and the central nervous system [6,7]. A recent paper has identified a novel arginine sensor that may play an important role in detecting intracellular nutritional status by activating the mechanistic target of rapamycin (mTORc1) pathway suggesting that arginine in particular may play a significant role in defining cellular health and viability [8]. Arginine has also been reported to play an important role in the maintenance of immune privilege in the cornea with inhibition of arginase activity associated with graft rejection [9].

Numerous studies have shown that arginine supplementation may increase wound healing following injury [10,11,12]. Oral administration of arginine has also been associated with improved wound healing following epidermal burns of the skin [13,14], as well as reduced incidence of necrotizing enterocolitis in premature infants following birth [15]. Further kinetic studies have identified increased arginine catabolism in burn victims suggesting that this amino acid may play a fundamental role in regulating tissue growth and repair [16,17,18]. These studies suggest that arginine may be an important metabolite involved in anabolic processes that are required for tissue regeneration following injury or diseases that lead to formation of a defective extracellular matrix (ECM). Whether arginine or nutritional status influence ECM deposition and tissue regeneration by resident cells remains an open question.

The cornea is an avascular tissue that provides two-thirds of the resolution power achieved in the human eye and requires nutrients provided from the aqueous humor and tear film. Collagen types I and V constitute the majority of the stromal ECM in the well-organized lamellar structure required for maintaining transparency and favorable biomechanical properties of the cornea that are essential for quality vision [19]. The influence of extracellular metabolite flux and nutritional status in regulating ECM deposition within the cornea is not well understood.

Keratoconus (KC) is a common corneal ectasia characterized by thinning of the central corneal apex which results in protrusion of the frontal region of the eye leading to a significant reduction in visual acuity [20]. Defects in ECM deposition by human keratoconus cells (HKCs) derived from the corneal stroma have been identified as a defining characteristic to the pathology of KC [21,22]. We have previously reported that HKCs have significantly downregulated cytosolic arginine compared to normal corneal keratocytes in 2D conventional cultures and deposit less ECM in 3D constructs [22,23]. As an extension of that work, our current study explored the effects of arginine supplementation on ECM secretion and metabolic flux in non-KC human corneal fibroblasts (HCFs) and HKCs cultured in a 3D in vitro stromal model to determine if increasing extracellular arginine levels can promote collagen secretion. We hypothesized that arginine supplementation would improve ECM secretion and deposition by HKCs by targeting a metabolic deficit of cytosolic arginine. Our findings suggest that arginine metabolism is an important pathway involved in collagen secretion by corneal fibroblasts that may potentially serve as a therapeutic target in the context of corneal thinning and keratoconus.

## 2. Materials and Methods

### 2.1. Isolation of Primary Human Corneal Fibroblasts

All experiments were completed with prior IRB approval from the University of Oklahoma Health Sciences Center (Protocol # 3450). The research adhered to the tenets of the Declaration of Helsinki. Primary human corneal fibroblasts were isolated from corneas derived from non-KC patients (HCFs) and KC patients (HKCs), as previously described [23,24,25]. Briefly, HCFs were isolated from human cadaver corneas of individuals with no prior history of ocular or corneal diseases (National Disease Research Interchange, Philadelphia, PA, USA). HKCs were isolated post-corneal transplantation from corneas of individuals with clinically diagnosed KC performed at Dean McGee Eye Institute (Oklahoma City, OK, USA). Upon tissue collection, the corneal epithelium and endothelium were removed from the stroma by mechanical scrapping with a sterile razor. The stromal tissue was cut into small pieces (~2 × 2 × 2 mm) and placed into small flasks, incubated for 10–15 min to allow adhesion of explant to flask followed by addition of Eagle’s Minimum Essential Medium (EMEM) (ATCC, Manassas, VA, USA) with 10% fetal bovine serum (Atlanta Biologicals, Lawrenceville, GA, USA) and 1× antibiotic–antimycotic supplement (contains penicillin (100 units/mL), streptomycin (100 µg/mL), and amphotericin B (250 ng/mL); Gibco, Life Technologies, Grand Island, NY, USA). Following 2–4 weeks of incubation at 37 °C/5% CO_2_, corneal fibroblasts migrated out of the tissue explant and were passaged into a T175 flask and grown to 80% confluence prior to seeding into constructs.

### 2.2. 3D In Vitro Model

3D constructs were assembled as described previously [22]. Briefly, 10^6^ HCFs or HKCs were seeded into each well in 6-well transwell plates containing a 0.4 μm pore polycarbonate membrane (24 mm transwell with 0.4 µm pore polycarbonate membrane insert, product # 3412, Corning Costar, Charlotte, NC, USA). A stable Vitamin C derivative (0.5 mM 2-O-α-D-glucopyranosyl-L-ascorbic acid, American Custom Chemicals Corporation, San Diego, CA, USA) in 10% FBS EMEM with 1× antibiotic–antimycotic was used to stimulate ECM secretion over a 4-week period with the media changed at least three times per week. Media was collected at week 4 in clean, sterile microcentrifuge tubes and immediately analyzed by Western blot. Arginine solutions (5, 10, and 15 mM) were prepared by dissolving L-arginine ((S)-2-amino-5-guanidinopentanoic acid, Sigma Aldrich, St. Louis, MO, USA) in complete EMEM media followed by pH correction and filter-sterilization (0.2 μm filter). The basal EMEM formulation included 0.6 mM of L-arginine·HCl (ATCC, Manassas, VA, USA).

### 2.3. Metabolite Extraction

Metabolites were isolated from cells as previously described. Briefly, constructs were collected, washed 3× with 1× phosphate-buffered solution, and incubated with ice-cold 80% methanol for 15 min at −80 °C. Cell lysate/methanol solution was then centrifuged at 14,000× *g* for 5 min at 4 °C. Resuspension of cell pellet was repeated 2× and samples combined and dried by a vacuum centrifuge (Eppendorf Vacufuge Concentrator, Eppendorf, Hamburg, Germany). Dried pellets were stored at −80 °C until further use.

### 2.4. Targeted Mass Spectrometry

Metabolite pellets were dissolved in high-performance liquid chromatography (HPLC)-grade water and analyzed for metabolite quantification using targeted microcapillary liquid chromatography-tandem mass spectrometry (LC-MS/MS) using a hybrid 5500 QTRAP triple quadrupole mass spectrometer (AB/SCIEX, Framingham, MA, USA) coupled to a Prominence UFLC system (Shimadzu, Kyoto, Japan) and analyzed with selected reaction monitoring (SRM) with positive/negative polarity switching. Label-free quantification via MultiQuant 2.1 software (AB/SCIEX, Framingham, MA, USA) was used to measure metabolite flux between samples, as previously described [23,26,27,28].

### 2.5. Real-Time Polymerase Chain Reaction (RT-PCR)

Constructs were isolated and total RNA was immediately extracted using TRIzol according to standard protocols (Ambion TRIzol Plus RNA Purification Kit, Life technologies, Carlsbad, CA, USA) [29]. cDNA was then synthesized (SuperScript III First-Strand Synthesis, Invitrogen, Carlsbad, CA, USA) and stored at −80 °C until further analysis. TaqMan probes for the housekeeping gene, glyceraldehyde-3-phosphate (GAPDH), and arginine-related genes (arginase (ARG1), eNOS (NOS3), iNOS (NOS2), and ornithine D (OAT)) were incubated with 10 ng of cDNA and addition of the Taqman Fast Advanced Master Mix (Applied Biosystems, Foster City, CA, USA) in separate reactions followed by analysis on a RT-PCR thermocycler (StepOnePlus RT-PCR, Applied Biosystems, Foster City, CA, USA).

### 2.6. Western Blot

Conditioned media was collected in sterile Eppendorf tubes and stored at −20 °C until further use. Cell lysates were isolated using 1× radioimmunoprecipitation assay (RIPA) buffer with a protease inhibitor cocktail (Sigma Aldrich, St. Louis, MO, USA) and incubated on ice for 30 min, followed by centrifugation at 12,000× *g* for 15 min at 4 °C to pellet cell debris. The clear supernatant was isolated and stored at −20 °C until further use. A bicinchoninic acid (BCA) assay (ThermoScientific, Rockford, IL, USA) was performed to determine total protein levels followed by Western blot analysis with at least 90 μg protein loaded into each well. Sodium dodecyl–sulfate polyacrylamide gel electrophoresis (SDS-PAGE) was performed using Tris-glycine gradient gels (4–20%) (Novex, Life technologies, Carlsbad, CA, USA) electrophoresed at 130 V for 1.5 h followed by transfer onto a nitrocellulose membrane (0.45 μm) (BioRad, Hercules, CA, USA) at 100 V for 1 h on ice. A Ponceau stain (Figure S3) was performed following transfer to show equal protein loading by incubating Ponceau S solution (0.1% (*w*/*v*) in 5% acetic acid, Sigma Aldrich, St. Louis, MO, USA) with membrane for 5 min, followed by washing with distilled water for 2–3 min. Blots were blocked in 5% dry milk or 5% bovine serum albumin (BSA, BP1605-100, Fisher, Fair Lawn, NJ, USA) for 1 h at room temperature with shaking. The following primary antibodies were prepared at a 1:1000 dilution in 1–2% BSA in Tris-buffered saline with 0.1% Tween 20 immediately prior to use: collagen type I (ab34710), collagen type III (ab7778), collagen type V (ab94673), fibronectin (ab2413), and GAPDH (ab9485) (Abcam, Cambridge, MA, USA). The fluorescent secondary (Alexa Fluor 568, donkey anti-rabbit, Life Technologies, Eugene, OR, USA) (1:2000) was incubated with the probed membrane for 1 h at room temperature with rocking followed by washing and imaging (UVP Gel Imaging System, Upland, CA, USA).

### 2.7. Statistical Analysis

All data was analyzed using GraphPad Prism (GraphPad Prism version 9.1.1 for Windows, GraphPad Software, San Diego, CA, USA). Statistical significance was determined using a two-way ANOVA with multiple comparisons. A *p*-value < 0.05 was considered statistically significant. All bar graphs are depicted showing mean ± standard deviation with the sample size designated in the figure legend.

## 3. Results

### 3.1. Arginine Metabolism in Corneal Fibroblasts

**Figure 1:**
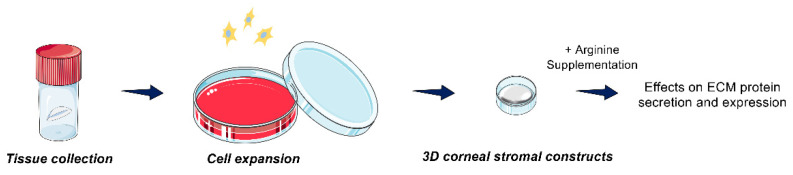
General experimental design for studying the effects of arginine supplementation on ECM secretion and expression by human corneal fibroblasts. The initial step was human corneal tissue collection, corneal fibroblast expansion, and construction of 3D corneal stromal models followed by arginine supplementation. Comparisons between healthy (HCF) and KC-derived (HKC) constructs were performed. Pictorials modified from Servier Medical Art under a Creative Commons Attribution 3.0 Unported License.

We previously applied a bottom-up tissue engineering approach [30] to construct 3D in vitro stromal constructs generated using non-KC (healthy control) and KC-derived primary human corneal fibroblasts [22,25,31]. To evaluate the effects of arginine supplementation in the context of KC, we generated control and KC 3D constructs that were maintained in culture for 4 weeks in the presence or absence of arginine supplementation (Figure 1 and Figure S1).

**Figure 2:**
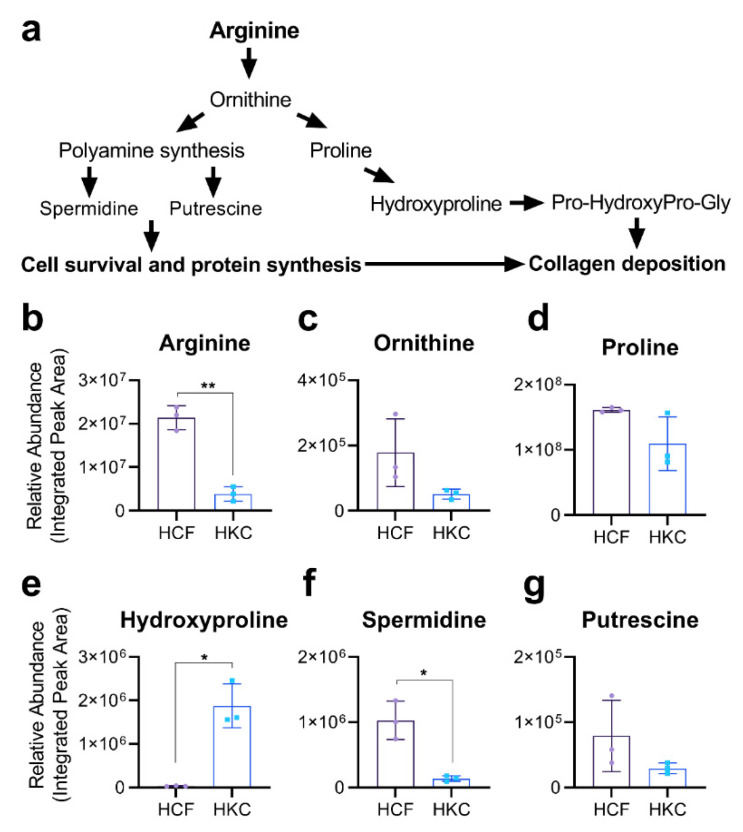
Arginine-related metabolite levels in HCFs and HKCs. (**a**) General schematic of arginine metabolism showing conversion of arginine to ornithine leading to either polyamine synthesis or generation of proline and hydroxyproline. (**b**–**g**) Metabolomics analysis of cytosolic levels of arginine-related metabolites (arginine and ornithine), collagen-related metabolites (proline and hydroxyproline), and polyamines (spermidine and putrescine) in 3D constructs of HCFs and HKCs. An unpaired, nonparametric *t*-test with Welch’s corrections was used to determine statistical significance with * *p* < 0.05 and ** *p* < 0.01 based on *n* = 3. Error bars represent standard deviation.

Arginine metabolism proceeds through ornithine, which can then be metabolized to the polyamines, spermidine and putrescine, or proline and hydroxyproline metabolites (Figure 2a). To determine if KC-derived corneal fibroblasts (HKCs) exhibited differential arginine metabolism compared to healthy controls (HCFs) when cultured in a 3D microenvironment in vitro, we utilized a LC-MS/MS metabolomics approach to determine the steady-state levels of arginine and arginine-related metabolites in HCF and HKC constructs (Figure 2b–g). Arginine levels were significantly lower in HKCs (5.5-fold, *p* = 0.0017) compared to control constructs with a similar trend in lower ornithine levels (3.5-fold, *p* = 0.16) (Figure 2b,c). Though proline levels were similar between HCFs and HKCs, hydroxyproline levels were notably lower in HCFs compared to HKCs (57-fold, *p* = 0.02, Figure 2e). The polyamine, spermidine, was present at higher basal levels in HCFs compared to HKCs (7.5-fold, *p* = 0.03) with no significant difference in putrescine levels detected (Figure 2f,g).

**Figure 3:**
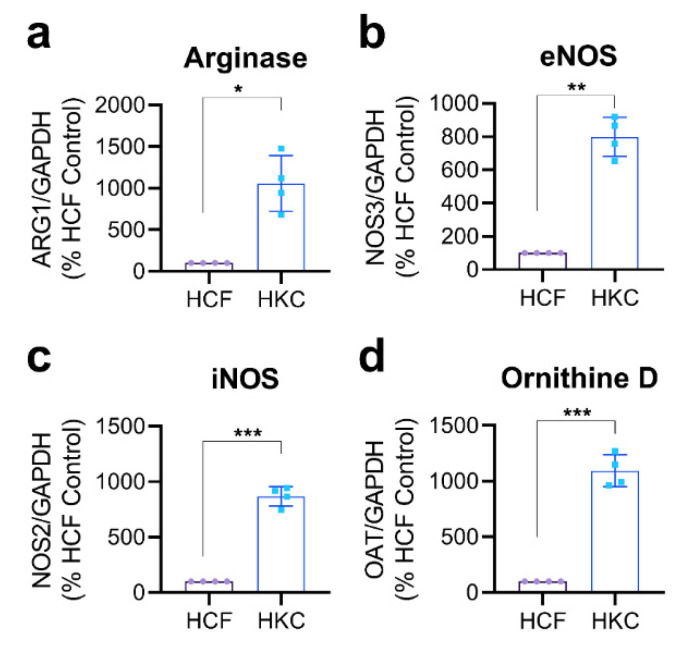
Gene expression patterns of arginine-related enzymes in HCFs and HKCs. Relative transcript levels of (**a**) arginase, (**b**) eNOS, (**c**) iNOS, and (**d**) ornithine D assessed by RT-PCR. Values shown as normalized to the housekeeping gene (GAPDH) and relative to HCF control levels. Statistical significance assessed using an unpaired, nonparametric *t*-test with Welch’s corrections with * *p* < 0.05, ** *p* < 0.01, and *** *p* < 0.001 based on *n* = 4. Error bars represent standard deviation.

To determine basal gene expression levels of proteins involved in arginine metabolism and/or downstream signaling, we isolated healthy (HCF) and HKC constructs and evaluated mRNA transcript levels of arginase, endothelial nitric oxide synthase (eNOS), inducible NOS (iNOS), and ornithine D relative to the housekeeping gene (glyceraldehyde 3-phosphate dehydrogenase, GAPDH) (Figure 3). We identified an overall increase in arginase (10.6-fold, *p* = 0.0107), eNOS (8-fold, *p* = 0.0013), iNOS (8.7-fold, *p* = 0.0004), and ornithine D (11-fold, *p* = 0.0008) in HKC constructs compared to their healthy counterparts (HCFs) (Figure 3). These results suggest that the lower arginine levels detected in HKCs may at least be partially attributed to differential arginine metabolism that influences arginine availability and downstream arginine-mediated signaling.

### 3.2. Arginine Uptake and Arginine-Related Metabolites

**Figure 4:**
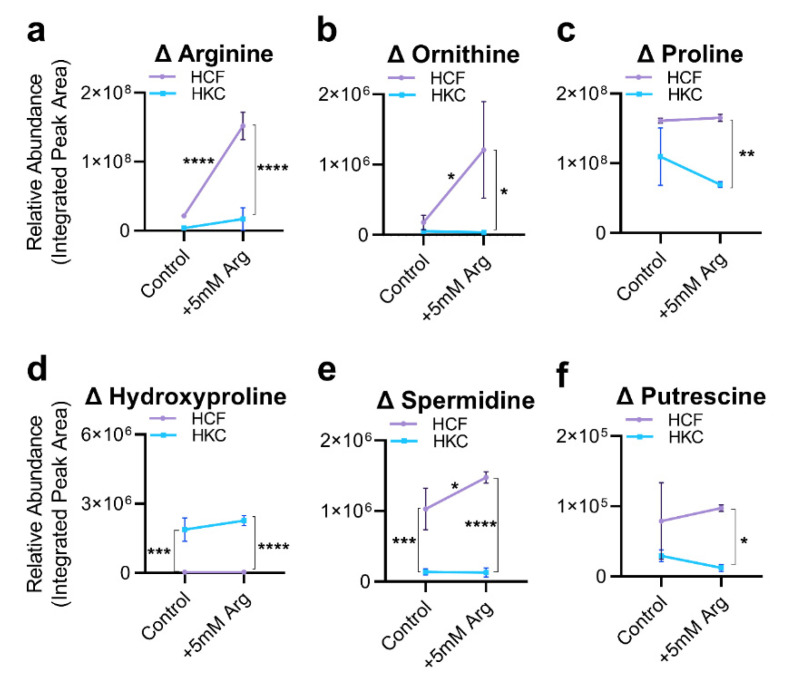
Arginine uptake and change in steady-state levels of arginine-related metabolites in HCFs and HKCs following arginine supplementation (5 mM). Relative abundance of (**a**) arginine, (**b**) ornithine, (**c**) proline, (**d**) hydroxyproline, (**e**) spermidine, and (**f**) putrescine in control and arginine supplemented (5 mM) constructs. Baseline control levels for these metabolites are also shown in Figure 2. Statistical significance assessed using a two-way ANOVA with Sidak’s multiple comparisons and * *p* < 0.05, ** *p* < 0.01, *** *p* < 0.001, and **** *p* < 0.0001 based on *n* = 3. Error bars represent standard deviation.

We next evaluated the effects of arginine supplementation on relative changes in arginine and arginine-related metabolites in healthy and HKC constructs. While basal cytosolic arginine levels were lower in HKCs than HCFs, supplementation with additional arginine (+5 mM) led to a large increase in arginine in healthy controls, with only a modest change in HKCs (Figure 4a). Likewise, a proportional increase in ornithine levels was observed in HCFs (11-fold, *p* = 0.027) with no significant change in HKCs (Figure 4b). While proline levels remained unchanged with arginine supplementation in both controls and HKCs, hydroxyproline levels remained high in HKCs with arginine supplementation (81-fold, *p* < 0.0001) (Figure 4c,d). Spermidine levels increased only in HCFs with arginine supplementation (1.4-fold, *p* = 0.034) with no change in putrescine levels in either HCFs or HKCs (Figure 4e,f).

### 3.3. Arginine Supplementation and ECM Secretion and Expression

**Figure 5:**
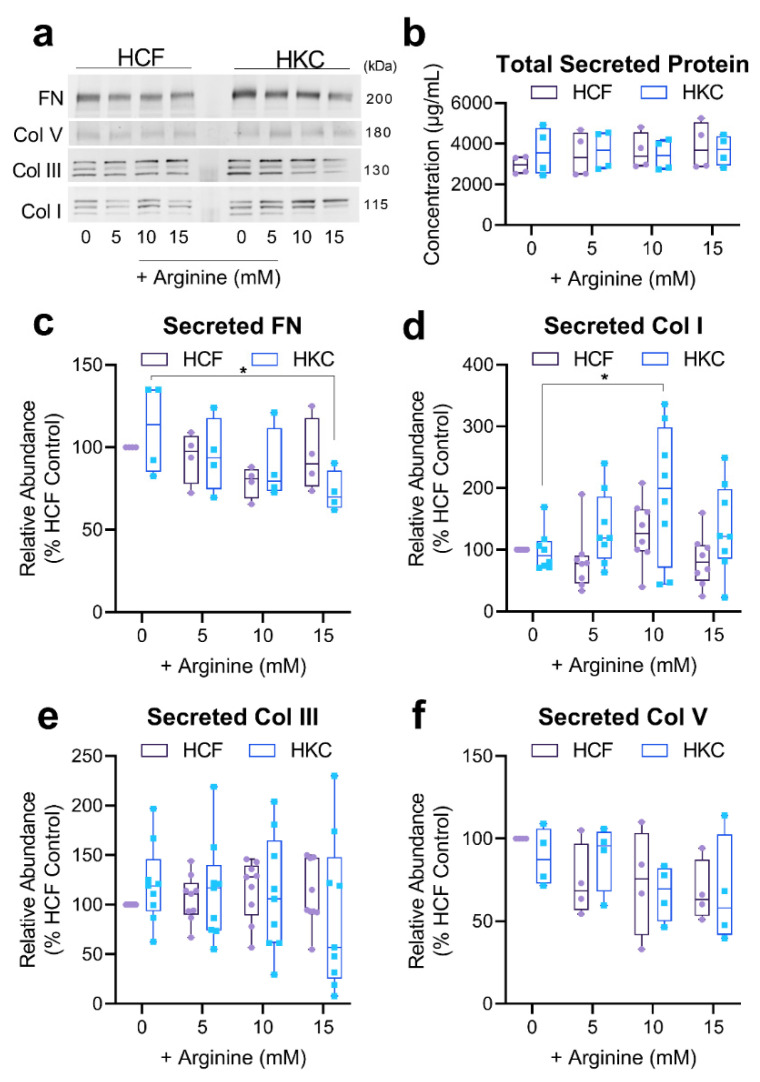
Secretion of matrix components by HCFs and HKCs in 3D constructs with increasing arginine supplementation. (**a**) Western blot of conditioned media isolated from 3D constructs seeded with HCFs and HKCs showing secretion of fibronectin (FN), collagen type I (Col I), collagen type III (Col III), and collagen type V (Col V) following supplementation of increasing concentrations of arginine at week 4. (**b**) Total secreted protein levels determined using a BCA assay. (**c**–**f**) Quantification of ECM proteins, FN, Col I, Col III, and Col V, found in conditioned media detected by Western blot with values normalized to the HCF control. Data are shown as box and whisker plots (minimum, first quartile, median, third quartile, and maximum) (*n* ≥ 4). A two-way ANOVA with Sidak’s multiple comparisons was used to determine statistical significance, with * representing *p* ≤ 0.05.

Since KC is associated with thinning of the stromal ECM, we sought to determine if arginine supplementation was a viable option for promoting favorable ECM deposition by HKCs. We measured fibronectin, and collagen types I, III, and V secretion in conditioned media analyzed by Western blot following supplementation with increasing concentration of arginine (5, 10, and 15 mM) (Figure 5a and Figure S4). As fibronectin and collagen are very distinct in terms of amino acid composition and structure [5,32], we sought to determine whether arginine supplementation influenced all ECM protein secretion indiscriminately or exhibited possible specificity for collagen expression. Arginine supplementation did not significantly modulate total secreted protein levels found in the culture medium of HCFs or HKCs (Figure 5b). Interestingly, high arginine concentrations of 15 mM significantly reduced fibronectin secretion (1.5-fold, *p* = 0.037) in HKCs with slight reductions in HCFs at 10 mM arginine supplementation (Figure 5c). Arginine supplementation (10 mM) also led to increased collagen type I secretion by 1.94-fold (*p* = 0.019) in HKC with no significant modulation in collagen type III or V secretion in HCFs or HKCs (Figure 5d–f). These results suggest that arginine supplementation favors collagen type I secretion over the other major isoforms (types III and V) expressed by human corneal fibroblasts.

**Figure 6:**
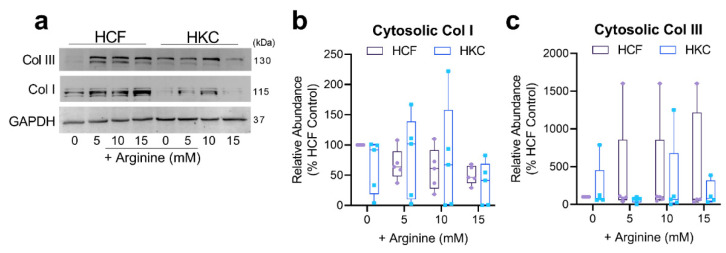
Cytosolic collagen protein expression. (**a**) Representative Western blots and (**b**,**c**) quantification of cytosolic collagen type I (Col I) and collagen type III (Col III) protein expression in HCFs and HKCs. Statistical significance evaluated based on a two-way ANOVA with Sidak’s multiple comparisons based on *n* > 3.

To determine if cytosolic levels of collagen type I and III were modulated by excess arginine, we probed cytoplasmic fractions of HCF and HKC constructs. In agreement with the effects of arginine supplementation on collagen secretion, cytosolic collagen type I showed an elevated trend in HKCs following 10 mM arginine supplementation with no significant differences in collagen type III protein expression in either HCFs or HKCs (Figure 6 and Figure S5). These results suggest that arginine supplementation may promote collagen expression by corneal fibroblast in vitro.

## 4. Discussion

As a naturally occurring amino acid, arginine supplementation is an attractive therapeutic option for increasing wound healing due to its extremely low toxicity, high availability, and low cost. A number of studies have shown improved tissue regeneration following arginine treatment [10,11] suggesting that arginine may regulate ECM deposition either through metabolic regulation or by promoting proliferation. To the authors’ knowledge, no studies have tested the effects of arginine in stimulating ECM secretion by corneal stromal fibroblasts in KC.

In order to distinguish which pathways of arginine metabolism play an important role in influencing collagen deposition, we utilized a metabolomics approach in this study to measure steady-state metabolite levels related to arginine and collagen-related metabolites. It is known that arginine metabolism primarily occurs via the urea cycle with conversion of arginine to ornithine, then to citrulline, and ultimately arginosuccinate and fumarate. In this study, we found that 5 mM arginine supplementation promoted elevated steady-state levels of cytosolic arginine and ornithine in healthy constructs. Interestingly, arginine supplementation did not appear to increase hydroxyproline levels in either HCFs or HKCs, but rather increased levels of the polyamine spermidine. With arginine levels increased, amounts of the spermidine were proportionally increased in HCFs. An alternative approach to increase endogenous arginine levels in HKCs might include a targeted inhibitor of arginase, the primary enzyme responsible for its conversion. A number of arginase inhibitors mainly for targeting endothelial cells and vascular dysfunction have been reported [33]. Further studies of the effects of L-norvaline and other selective arginase inhibitors on ECM deposition in fibroblasts might provide insight into whether this pathway can be targeted therapeutically to improve wound healing. Increasing proline or related precursors might also influence collagen expression and wound healing responses [34,35,36] but has remained relatively unexplored in the context of corneal biology.

The polyamine pathway is known to be important in regulating DNA replication, proliferation, and cell survival [4,37]. Spermidine and putrescine are polyamines important in regulating DNA synthesis [38] and are derived from arginine via ornithine catalyzed by ornithine decarboxylase [39], as well as from conversion of arginine to agmatine [40,41]. However, it is unclear if these metabolites modulate collagen deposition directly or indirectly by favoring downstream pro-survival mechanisms. In addition to the urea cycle, other metabolites may also be contributing to arginine flux within the cell, such as creatine and glutamate, which may be influencing the differential cellular responses to excess arginine observed between HCFs and HKCs in our study. A limitation of our study was the single concentration of arginine (5 mM) included in our metabolomics study, and thus, additional studies testing a dose-response of arginine supplementation on steady-state levels of collagen-related metabolites are warranted.

Previous studies have shown a pH-dependence of arginase II, which is involved in metabolism of arginine to ornithine [42,43]. Though we did not detect significant increases in proline or hydroxyproline with arginine supplementation, collagen type I secretion was significantly increased in HKCs by 10 mM arginine supplementation suggesting that arginine-mediated ECM secretion may not involve increased conversion of arginine to the amino acids, proline and hydroxyproline, which comprise ~33% of collagen structure [36]. The slight differences in molecular weight of collagen sub-types assessed in our study between predicted molecular weights may be attributed to differences in post-translational modifications, pH of the microenvironment, and effectiveness of the denaturing and reducing conditions, among other factors, which may influence the observed molecular weight detected by Western blot. Our study provides evidence to suggest that arginine supplementation may support increased collagen type I secretion by HKCs, but the biochemical pathway involved in this observed effect is unclear. Moreover, further studies are required to show that the collagen secreted by HKCs is properly assembled and integrated into collagen fibrils within the stromal ECM and determine whether arginine supplementation can overcome the inherent defects present in HKCs that appear to promote stromal thinning in the KC cornea.

## Data Availability

All relevant and supporting data are contained within the manuscript and available from the corresponding author upon reasonable request.

## Supplementary Materials

The following are available online at https://www.mdpi.com/article/10.3390/cells10082076/s1, Figure S1: Brightfield image of primary human corneal fibroblasts cultured in 2D conventional culture. Scale bar = 200 µm, Figure S2: Preliminary study testing the effects of arginine supplementation in 2D conventional cultures, Figure S3: Ponceau staining of a western blot analysis of conditioned media showing relatively equal loading between sample, Figure S4: Uncropped blots of proteins measured by Western blot shown in Figure 5, Figure S5: Uncropped blots of proteins measured by Western blot shown in Figure 6. Membranes were cut following blocking and prior to probing with antibody.
