# Supplementary material for: Arginine Supplementation Promotes Extracellular Matrix and Metabolic Changes in Keratoconus
Source: Cells. 2021 Aug 13;10(8):2076. doi: 10.3390/cells10082076 (PMC8394349; doi:10.3390/cells10082076)
Supplement: Supplementary file 1 [file cells-10-02076-s001.zip › cells-1327559-SI.pdf]

**Supplemental Materials**

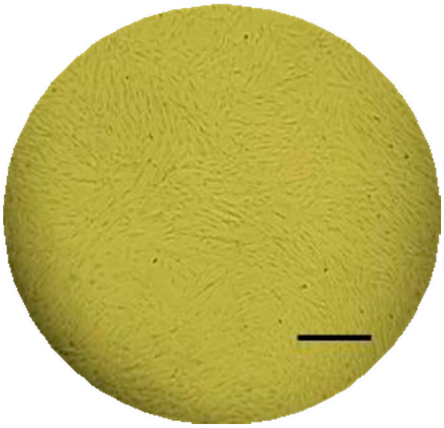

**Figure S1.** Brightfield image of primary human corneal fibroblasts cultured in 2D conventional culture. Scale bar = 200 μm.

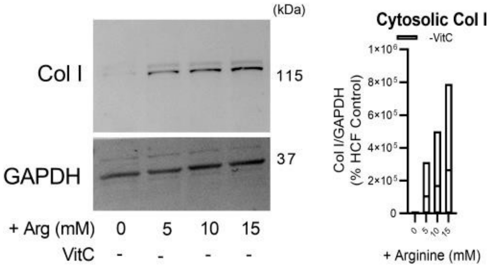


**Figure S2.** Preliminary study testing the effects of arginine supplementation in 2D conventional cultures. Primary human corneal fibroblasts were seeded in 6-well plates containing EMEM with 10% FBS and 1X antibiotic/antimycotic ($10^6$ cells/well). At t= 24 hours post-seeding, arginine- supplemented media was added, and the cells were cultured for an additional 1-2 weeks followed by cell lysis, protein isolation, and Western blot analysis. Representative western blots (*left panel*) and quantification(*right panel*) shown for Collagen type I expression normalized to GAPDH (n=3).

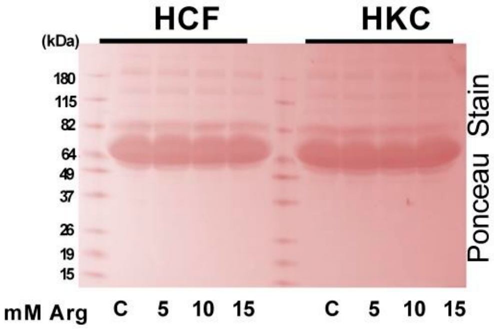


**Figure S3.** Ponceau staining of a western blot analysis of conditioned mediashowing relatively equal loading between sample.

Probed with anti-FN
(ab2413, 1:1000, Abcam)

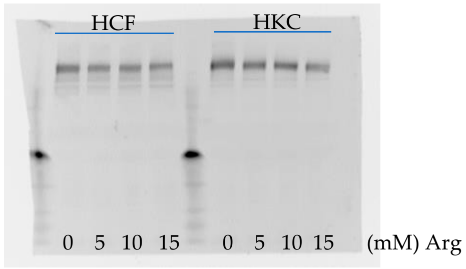


Probed with anti-Col V
(ab94673, 1:1000, Abcam)

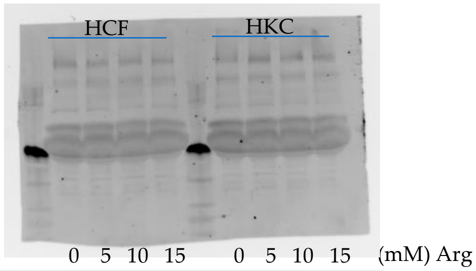


Probed with anti-Col III
(ab7778, 1:1000, Abcam)

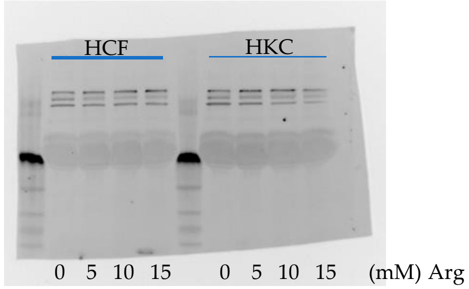


Probed with anti-Col I
(ab34710, 1:1000, Abcam)

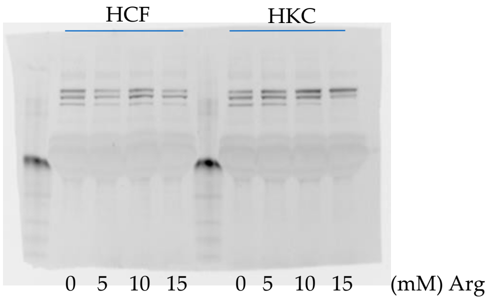


**Figure S4.** Uncropped blots of proteins measured by Western blot shown in Figure 5.

Probed with anti-Col III
(ab7778, 1:1000, Abcam)

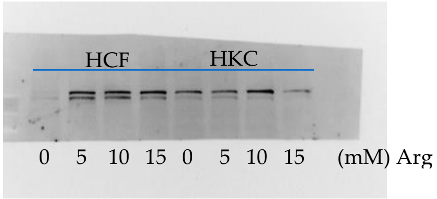


Probed with anti-Col I
(ab34710, 1:1000, Abcam)

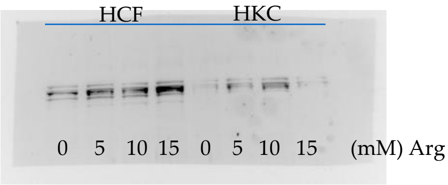


Probed with anti-GAPDH
(ab9485, 1:1000, Abcam)

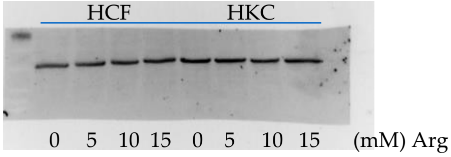


**Figure S5.** Uncropped blots of proteins measured by Western blot shown in Figure 6.Membranes were cut following blocking and prior to probing with antibody.
